# Adaptive Genomic Variation Across Sub‐Alpine Climates in a Keystone Forest Tree: Implications for Seed Sourcing for Restoration in a Changing Climate

**DOI:** 10.1111/eva.70339

**Published:** 2026-11-06

**Authors:** John W. Whale, Katarina C. Stuart, Jessica Bruce, Melinda Pickup, David L. Field

**Affiliations:** ^1^ Applied Biosciences Macquarie University Macquarie Park New South Wales Australia; ^2^ School of Science Edith Cowan University Joondalup WA Australia; ^3^ Greening Australia, Native Seed Centre for Restoration Western Sydney University Richmond New South Wales Australia

## Abstract

A major challenge for ecological restoration is seed sourcing decisions that ensure that restored populations have the adaptive variation for current and future environments. This is increasingly important for alpine and sub‐alpine communities where climate change is predicted to be rapid and drive shifts in selective pressures. Selecting source populations representing future climates is an evolutionary‐based strategy for increasing adaptive capacity, but requires an understanding of population genetic structure, identification of putatively adaptive genomic variation and an assessment of how these change across the landscape. Using 3978 single‐nucleotide polymorphism (SNP) markers we assessed the population structure and diversity of 33 sampled populations of *Eucalyptus delegatensis* from across its distribution, including 22 populations from the mainland ( *E. delegatensis* subsp. *delegatensis*) and 11 from Tasmania ( *E. delegatensis* subsp. *tasmaniensis*). Genotype‐environment association (GEA) analysis identified strong genetic associations with seasonal climate variables, particularly minimum temperature of the coldest month, and revealed a parallel cline at one outlier locus across the mainland and Tasmanian range, consistent with independent sorting of standing variation along environmental gradients in both subspecies. By projecting genetic offset under future climate change scenarios, we identify regions of the species' range most vulnerable to projected change; despite this, seed‐sourcing projections suggest that suitable future climate matches may be available for most of the range. Together, these findings demonstrate the value of comparative GEA approaches in validating outlier loci, and show that even in regions of projected dramatic environmental shifts, such as Australian alpine environments, landscape genomic analyses can help identify potential suitable seed sources for climate‐resilient ecological restoration.

## 1 Introduction

Understanding plant adaptation to the environment is not only fundamental to characterising adaptive variation but is also critical to managing species in a changing climate. For ecological restoration, information on patterns of adaptation to current and future conditions is central to seed sourcing decisions (Havens et al. 2015). The paradigm of using local seed sources has historically dominated provenance choice for restoration (Hufford and Mazer 2003; Broadhurst et al. 2008), based on the premise that genetic variation within populations is associated with adaptation to their local environment, including climate and soils, and that these environmental variables scale predictably with geographic distance (Leimu and Fischer 2008). This consideration is especially relevant in alpine and sub‐alpine landscapes, where climate change is predicted to be rapid and drive large‐scale ecological change (Dainese et al. 2024; Davey et al. 2024), particularly for long‐lived species that cannot migrate or adapt at a comparable pace.

However, restricting potential seed sources to populations that are geographically close (local seed sources) may not provide the optimum genetic composition for restoration. If local source populations are fragmented and small, they may be inbred and contain reduced adaptive genetic diversity (Weeks et al. 2011). While avoiding local provenances with low adaptive potential that may be maladapted to future climates, geographically distant sources are also not guaranteed to carry suitable genetic variability. Low overall genetic differentiation (e.g., low *F*_ST_) between populations does not necessarily indicate similar frequencies at loci underlying climate resilience, since climate adaptation is more often driven by subtle changes in allele frequencies at shared loci among populations across the species distribution rather than the presence of population‐specific novel mutations (Ahrens et al. 2019; De La Torre et al. 2019). Implementing effective seed sourcing strategies therefore requires an understanding not only of population differentiation, but the frequency of climate‐relevant adaptive alleles across the landscape.

Alpine and sub‐alpine species inhabit environments characterized by steep gradients in altitude, temperature, snow cover, and precipitation, making them particularly vulnerable to global environmental change. Traits associated with cold adaptation, such as cold stratification requirements and frost tolerance, are likely to be particularly sensitive to the rapid warming projected for these environments. Beyond changes in temperature and precipitation, alpine regions are also expected to experience increased fire frequency under future climate projections (Boer et al. 2020; Collins et al. 2022; Wilson et al. 2021), imposing additional selective pressures on traits related to fire resilience, including bark thickness, length of juvenile periods, and resprouting capacity (Enright et al. 2014; Gallagher et al. 2021; Pausas and Bradstock 2007). Together, these concurrent shifts in climate and fire frequency will intensify selection on existing adaptive variation, increasing the risk of population decline in areas where adaptive variation is limited or constrained. In this context, climate‐adjusted seed sourcing (Hoffmann et al. 2015; Prober et al. 2015) is likely to be an important risk‐based evolutionary strategy for restoring fire‐prone alpine and sub‐alpine species in areas where increased aridity and seasonal changes in temperature and precipitation are predicted. In Australia, alpine and sub‐alpine communities are especially at risk due to their high levels of endemism and limited geographic extent, representing only a small proportion of the continent. Eucalypts dominate these forests in sub‐alpine environments, and are well known to show evidence of local adaptation in association with environmental variables such as temperature, aridity, isothermality, and precipitation in other environments on the Australian continent (Supple et al. 2018, Buttler et al. 2022, Felipe et al. 2022). However, relatively few studies have examined local adaptation of eucalypt species in sub‐alpine environments in Australia, leaving a critical gap in knowledge for keystone taxa in these ecosystems.

The field of landscape genomics has expanded rapidly over the past decade, with the development of statistical approaches allowing identification of candidate genes involved in environmental adaptation (Bragg et al. 2015). Among these, genotype‐environment association (GEA) analyses leverage genome‐wide single nucleotide polymorphisms (SNPs) to identify which genetic differences between populations are associated with environmental variables that characterize the population of origin (Caye et al. 2019; Gautier 2015). However, a central challenge in landscape genetics is distinguishing signals of local adaptation from demography and spatial autocorrelation. Validation of outlier loci often requires testing variant fitness under field conditions, which is often impractical for species such as long‐lived trees. An alternative and powerful approach comes from testing for parallel geographic clines for putative adaptive variants across independent lineages experiencing similar environmental gradients (Lotterhos and Whitlock 2015). Even when lineages share ancestral genetic variation, as is expected between sister species or subspecies, concordant clines in allele frequencies along environmental gradients in geographically isolated regions provide strong evidence of independent sorting of standing genetic variation by natural selection, rather than isolation by distance alone (Barrett and Schluter 2008). Demonstrated parallel clines therefore provide a valuable means of testing for adaptation in environmentally and spatially structured lineages.

This study investigated the patterns of local adaptation across climatic gradients in two subspecies of *Eucalyptus delegatensis* (Alpine Ash) in south‐eastern Australia, with the aim of identifying seed sourcing strategies that maximize adaptive capacity under current and future environmental conditions. *Eucalyptus delegatensis* is a keystone forest tree of mountainous regions in south‐eastern Australia and provides an ideal system to test hypotheses related to adaptation to sub‐alpine environments. The species comprises two recognized subspecies: *E. delegatensis* subsp. *delegatensis*, which is distributed across south‐eastern Victoria and New South Wales, and *E. delegatensis* subsp. *tasmaniensis*, which is restricted to Tasmania. The species occurs in cool temperate climates and is frost and snow tolerant, requiring cold stratification for germination (Horne and Robinson 1990) and has been susceptible to dieback and/or mortality events due to extreme climatic events (Ellis 1964; Liu and Kirkpatrick 2023). Previous ecological studies in *E. delegatensis* have shown that regeneration is influenced more by precipitation than temperature, with autumn rainfall the most important seasonal climate factor (Singh et al. 2021). On the mainland, it is distributed across a range of climatic conditions, from hotter and drier populations in the north and southeast of the range (NSW/Victoria) to the cooler and wetter regions in central Victoria. In Tasmania, the populations span similar environmental gradients, making this species particularly well suited to examine parallel climatic drivers of adaptation. Across its range, this species and associated vegetation communities provide critical ecosystem services including habitat provision, carbon sequestration and water quality. Despite its ecological importance, it is a species that is highly vulnerable to both drought (Singh et al. 2021) and increased fire frequency and intensity (McColl‐Gausden et al. 2021) under climate change.

Here, we use genome‐wide SNPs from populations across the geographic range of *E. delegatensis* and climatic and environmental variables to identify putatively adaptive genomic variation. We first examine genome‐wide SNPs across 33 populations of *E. delegatensis* to understand how genetic diversity and structure of populations are partitioned across the species range, focusing on the mainland populations ( *E. delegatensis* subsp. *delegatensis*), but also comparing these to populations from Tasmania ( *E. delegatensis* subsp. *tasmaniensis*). We then test whether: (1) populations show signals of local adaptation to climate, (2) minimum temperature shows the strongest signals of local adaptation, and (3) the same putatively adaptive loci show independent sorting across the same environmental gradients in both subspecies (mainland vs. Tasmania). Finally, we examine the turnover of alleles across the distribution of this species, associations with gene ontology (GO) function, and evaluate their implications for seed sourcing strategies in restoration.

## 2 Methods

### 2.1 Study Species and Sampling/Seed Sourcing

*Eucalyptus delegatensis* R.T. Baker is a keystone tree species that belongs in the subgenus *Eucalyptus*, section *Cineraceae* series *Fraxinales*. The species is a tall tree generally up to 50 m but can reach 90 m (Boland and Brooker 2006) and is endemic to mountainous regions of southeastern Australia. The primary differences between the subspecies are found in bark characteristics, juvenile leaf morphology and strategies for fire recovery. On the mainland, *E. delegatensis* subsp. *delegatensis* is considered an obligate seeder that does not regenerate after fire (primary juvenile period: 6–20 years; Fagg et al. 2013; Doherty et al. 2017), whereas *E. delegatensis* subsp. *tasmaniensis* regenerates both from seed and through epicormic resprouting, a trait that is only present in the Tasmanian subspecies (Rodriguez‐Cubillo et al. 2020). Recently, mainland *E. delegatensis* has been classified as endangered at the federal level with projections estimating a minimum range contraction of 49% over the next 100 years (Hammond et al. 2026).

*Eucalyptus delegatensis* seedlots from 33 provenances across the geographic range of both subspecies (Figure 1, Table 1) were sourced from the CSIRO Tree Seed Centre and Vic Forests. Seeds were first treated with 1% sodium hypochlorite bleach solution for 10 min (for quarantine purposes) then rinsed with water and dried before a 21‐day cold stratification period at 2°C to induce germination (Close and Wilson 2002). Following cold stratification, 1500 individuals were planted on petri dishes with moist filter paper for germination, and transplanted at ~4 weeks into 1‐l pots filled with 1:1 Osmocote Native Mix and RICHGRO Play Sand (Bunnings Burnley, VIC) with Osmocote slow‐release native fertilizer mixed in at field rate in a glasshouse at Edith Cowan University, Joondalup, Western Australia. Once seedlings had several true juvenile leaves, a total of 644 unique individuals were harvested and leaves freeze‐dried.

**FIGURE 1:**
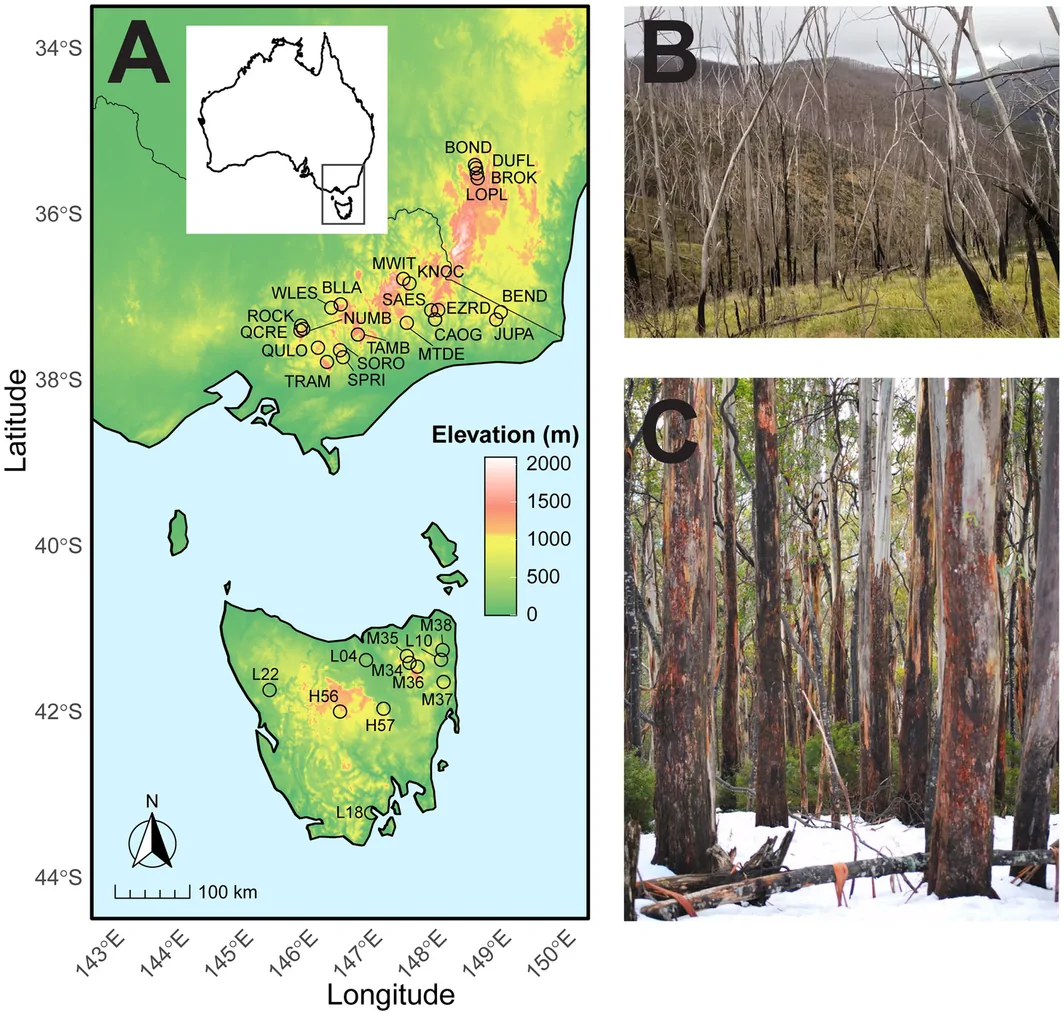
Sampled *E. delegatensis* populations (circles) in southeastern Australia (inset). Panel (A) shows a map of the elevational changes across the species distribution. Panel (B) shows unhealthy trees and panel (C) shows a healthy *E. delegatensis* stand. Photo credit to Thomas Fairman.

**TABLE 1:** Populations of mainland *E. delegatensis* with their location (Latitude, Longitude) and population genetic diversity statistics. Populations are arranged from northeast to southwest.

Location	Population	Latitude	Longitude	*n*	Ho	uHe	*F*is
Bondo State Forest	BOND^a^, ^b^	−35.413818	148.581906	13	0.097	0.130	0.211
Dubbo Flats	DUFL^a^, ^b^	−35.452246	148.599146	40	0.090	0.136	0.280
Broken Cart Trail	BROK^a^, ^b^	−35.515186	148.610762	13	0.095	0.131	0.231
Long Plains Road	LOPL^a^, ^c^	−35.574408	148.623881	11	0.101	0.135	0.201
Bendoc	BEND^b^	−37.187375	148.982829	30	0.116	0.163	0.257
Julies Patch	JUPA^b^	−37.277786	148.910761	30	0.115	0.167	0.283
Ezards Track	EZRD^c^	−37.162449	148.010929	12	0.095	0.138	0.255
Sawpit/Escarpment	SAES^c^	−37.165374	147.903316	17	0.090	0.135	0.265
Camp Owen Gap	CAOG^c^	−37.276662	147.964426	20	0.088	0.135	0.282
The Knocker	KNOC^c^	−36.842047	147.566396	20	0.110	0.150	0.227
Mount Wills Track	MWIT^c^	−36.787430	147.470672	17	0.095	0.138	0.259
Mount Delusion	MTDE^c^	−37.317430	147.524800	14	0.096	0.137	0.240
Tamboritha	TAMB^c^	−37.457959	146.765637	35	0.102	0.140	0.231
Black Landing	BLLA^c^	−37.094804	146.498423	9	0.108	0.135	0.169
Wales	WLES^c^	−37.135741	146.353972	25	0.109	0.146	0.205
South Road	SORO^c^	−37.647795	146.489128	6	0.097	0.130	0.202
Springs Road	SPRI^b^	−37.731428	146.534720	12	0.117	0.151	0.188
Rocksteady	ROCK^c^	−37.350692	145.886880	11	0.108	0.141	0.189
Number 5 Track	NUMB^c^	−37.390417	145.918228	19	0.104	0.136	0.197
Quartz Creek East	QCRE^c^	−37.409698	145.873985	20	0.103	0.139	0.205
Quiche Lorraine	QULO^c^	−37.617216	146.146778	17	0.095	0.139	0.257
Tramline C #5	TRAM^c^	−37.788603	146.286764	18	0.100	0.140	0.235

Abbreviations: *F*is, inbreeding coefficient; Ho, observed heterozygosity; *n*, number of individuals; uHe, unweighted expected heterozygosity.

^a^ Denotes provenances/populations are New South Wales origin, all other provenances are of a Victoria origin.

^b^ Denotes provenance is situated within the South Eastern Highlands Interim Biogeographic Regionalisation for Australia (IBRA) bioregion.

^c^ Denotes provenance within the Australian Alps IBRA bioregion.

### 2.2 SNP Generation and Bioinformatics

Harvested individuals were submitted to Diversity Arrays Technologies Pty Ltd. (Canberra, ACT, Australia) and sequenced using the DArTseq protocol (Kilian et al. 2012; Sansaloni et al. 2011) using *Pst*I and *Hpa*II restriction enzymes to generate a reduced‐genome complexity dataset. The *E. delegatensis* samples underwent reference free SNP calling following the DArTseq protocol, which uses a combination of de‐novo loci calling as well as closely related species mapping. In this instance the alignments were done to two closely related *Eucalyptus* reference genomes that either coexist or are also endemic to Australian sub‐alpine environments: *E. regnans* (accession #: ASM1418285v1, GenBank) and *E. pauciflora* (accession #: ASM 766332v1, GenBank). Loci were filtered using the DARTRVERSE v1.0.6 package (Gruber et al. 2018; Mijangos et al. 2022) in *R* (R Core Team 2024). The minimum missing data threshold was set to 40% per locus and 25% per individual, and loci were removed from the dataset if they were monomorphic, did not meet a read depth threshold of 10, and a reproducibility threshold of 0.99. One locus per 69 bp sequencing read was randomly retained, a minor allele frequency (maf) threshold was set to 0.02, and finally, a total of 27 loci under high linkage disequilibrium (> 0.5) were also removed. We chose to remove these loci from all analyses to avoid disproportionate weighting of the turnover signal toward randomly over‐sampled LD clusters arising from the reduced‐representation approach. After filtering, 409 individuals among 22 populations were retained for analyses. Additionally, the level of kinship within populations was assessed by running self‐coancestry analysis using the *beta.dosage* function in the HIERFSTAT v0.5–11 package (Goudet 2005; de Meeûs and Goudet 2007). This analysis identified few closely related individuals within the dataset, and given their low frequency, these were unlikely to meaningfully influence downstream results; relatedness filtering was therefore not deemed necessary (Figure S1).

### 2.3 Population Diversity, Differentiation and Structure

First, we aimed to assess patterns of genetic differentiation across the *E. delegatensis* range, examining population structure between the mainland and Tasmanian populations (the latter considered a subspecies), as well as structure within each region.

Population genetic structure analysis was performed using the LEA v3.16.0 package (Frichot and François 2015) by using Sparse Non‐negative Matrix Factorization (SNMF) method to estimate ancestry coefficients. SNMF was run for each *k*‐value between 1 and 22, with each *k*‐value run 12 times with 10 iterations. The cross‐entropy was inspected to determine the optimal *k*‐value. The optimal *k*‐value across each of the 12 runs was combined and estimated using the software CLUMPP v1.1.2 (Jakobsson and Rosenberg 2007), and graphical parameters drawn in DISTRUCT v1.1 (Rosenberg 2004). This analysis was done on the full dataset, and then repeated on the mainland only and Tasmania only dataset.

A discriminant analysis of principal components (DAPC, Jombart et al. 2010) was performed to further investigate the population structure relationships of *E. delegatensis* . We used the *find. clusters* function to estimate the number of genetic clusters within the dataset using ADEGENET v2.1.11 (Jombart 2008; Jombart and Ahmed 2011). The number of clusters was inspected for the lowest Bayesian Information Criterion (BIC) score. The *dapc* function was used to visualize population structure and clustering by transforming the data through principal component analysis (PCA) followed by the determination of clusters using discriminant analysis. We retained 195 principal components and two discriminant functions representing 70.0% of the variation and visualized as a 2D scatterplot. This analysis was done on the full dataset, and then repeated on the mainland only dataset.

Because of the strong genetic differentiation apparent between mainland and Tasmanian populations of *E. delegatensis* (see Section 3), we proceeded to conduct the remainder of our analyses on only the mainland population, unless otherwise noted, filtering the dataset into separate mainland and Tasmania datasets where both were refiltered to a 0.02 maf threshold.

Next, across all mainland sampling locations, we calculated the genetic diversity indices of observed (Ho), expected (He), unbiased expected heterozygosity (uHe), and inbreeding coefficient (*F*is), using the *gl.report.heterozygosity* function in DARTR. Genetic differentiation among populations was examined using global *F*_ST_, calculated using the Weir and Cockerham *F*‐statistic method (Weir and Cockerham 1984) in HIERFSTAT, while pairwise *F*_ST_ values were calculated between all populations using the *gl.fst.pop* function and a confidence interval of 0.95 in DARTR. Analysis of molecular variance (AMOVA) was calculated to estimate the genetic variance within and among populations partitioned by Interim Biogeographic Regionalisation for Australia (IBRA) regions (South Eastern Highlands and Australian Alps) using the *poppr.amova* function and 999 permutations in POPPR v2.9.6 (Kamvar et al. 2014) with significance levels calculated using *randtest* in ADE4 v1.7–22 (Chessel et al. 2004).

### 2.4 Influence of Geographic and Environmental Distance on Genetic Differentiation

Next, we sought to identify how much genetic differentiation could be attributed to geographic (spatial) versus climatic (environmental) attributes. All analyses in this section was conducted in *R*. First, we identified key climate variables; temperature and precipitation variables were downloaded from the 19 BIOCLIM variables in the WORLDCLIM v2.0 database (Fick and Hijmans 2017) using the *worldclim_global* function using the GEODATA v0.6–2 package at a 30s‐arc resolution. Climate data for each population was extracted using the TERRA v1.7–78 package (Hijmans et al. 2023) based on the GPS coordinates for the populations. To calculate mean annual heat moisture index (AHMI, Nitschke et al. 2017), we used mean annual temperature (T_MA_) and mean annual precipitation based on Bureau of Meteorology data from 1986 to 2005 using the formula AHMI = (T_MA_ + 10)/(P_MA_/1000). A Pearson's correlation coefficient (*r*) matrix was calculated between each of the 19 bioclimatic variables using the *cor* function. If the *r* was > ±0.7 between two variables, these were considered strongly correlated, and a leave‐one‐out approach was used to reduce co‐linearity across the retained environmental variables, with the retained variables informed by those of known biological importance for *E. delegatensis* . A total of eight climate variables, covering annual, seasonal, and extreme variables, were selected: mean annual temperature (BIO01, T_MA_), mean temperature seasonality (BIO04, T_SN_), mean minimum temperature of the coldest month (BIO06, T_MIN_), mean temperature of the driest quarter (BIO09, T_DryQ_), mean annual precipitation (BIO12, P_MA_), mean precipitation of the driest month (BIO14, P_MIN_), mean precipitation of the warmest quarter (BIO18, P_WarmQ_), and AHMI. Of these climate variables, only T_SN_ and T_MIN_ exhibited a *r* > 0.7.

To estimate the association between genetic variation, geographic distance, and climate, a multiple matrix regression with randomisation (MMRR) analysis was conducted (Wang 2013). MMRR allows for the partitioning of the relative amount of genetic differentiation explained between sampling locations that can be attributed to geographic distance or environmental dissimilarity. For this, three matrices were produced, with each pairwise comparison between sampling locations represented by a single value in each matrix. Firstly, a genetic matrix was produced using the function stamppNeisD in the STAMPP v1.6.3 (Pembleton et al. 2013). The function *distm* (distance function = distHaversine) in the GEOSPHERE v1.5–20 (Hijmans 2024) package was used to produce a spatial distance matrix for the sampling locations' latitude and longitude values. Finally, the eight environmental variable values for the sampling locations were computed into a distance matrix using the STATS (R Core team 2024) function *dist* (method = “euclidean”). MMRR analysis was then used to identify the explanatory power that the spatial and environmental matrices had on the genetic matrix, performing 9999 permutations.

### 2.5 Genotype‐Environment Associations

Next, for the mainland populations, we sought to identify climate‐'associated loci of *E. delegatensis using* three genotype‐environment association (GEA) approaches: regression based latent factor mixed models implemented through LFMM2 (Caye et al. 2019), constrained multivariate ordination approach using partial redundancy analysis (RDA, Forester et al. 2018), and Bayesian hierarchical modeling implemented through BAYPASS v3.0 (Gautier 2015). Each of these three approaches accounts for population structure in different ways and identifies environmentally associated loci using different biological data (individual or population level) and statistical criteria. To maximize retention of true positives, we adopted a conservative‐inclusive strategy by retaining the union of SNPs identified across analytical approaches. All analyses incorporated the eight climate variables described above. Putative environmentally associated loci will be validated by future analyses.

LFMM2 (Caye et al. 2019) is a univariate method that requires a genetic dataset with no missing information. Analysis for this GEA method was run in R. Missing SNPs were thus imputed using the *impute* function using the median method across all populations to produce a complete genotype matrix. Population structure was accounted for by performing a PCA and the interpretation of *K* confirmed by using the broken stick criterion. This *K* estimate was used in the *lfmm_ridge* function in LFMM v1.1 package to fit the regression model to the number of *K* factors. Each environmental variable was individually placed within the model using the *lfmm_test* function and *p*‐values calibrated using the genomic inflation factor control method. A false discovery rate threshold of *α* = 0.001 was applied to limit the detection of false positives.

Partial RDA is a multivariate approach that allows for the canonical relationships between allele frequencies and environmental predictors to be found, while taking into account the confounding effects of geography through the inclusion of a second set of conditioning distance variables. To avoid the need for imputation, we conducted this analysis on population level data, using PLINK v1.9 (Purcell et al. 2007) to calculate population allele frequencies, before analysis was then continued in R. The spatial distance matrix produced in the MMRR analysis above was processed through the *pcnm* function in VEGAN v2.6–8 (Oksanen et al. 2019), and the first 3 axes retained, which would serve as the conditioning variables to account for spatial structure within the allele frequency data. The *rda* function in VEGAN was used to perform a partial RDA, with the allele frequencies as the dependent variables, the populations' environmental variables as the explanatory constraining matrix, and the top 3 geographical *pcnm* variables as the conditioning spatial variables. The significance of the RDA model was assessed using the VEGAN function *anova.cca* which determined the overall model to be significant. Finally, we identified environmentally associated loci as SNPs that loaded more than 2.5 standard deviations from the mean for each of the first three RDA axes. Because RDA cannot determine on its own which environmental variable most strongly predicts a specific SNPs' allele frequencies, we regressed all identified SNPs against the eight environmental predictors and assigned the SNP to the one that had the highest correlation using the *cor* function in the STATS package (R Core Team 2024).

BAYPASS uses Bayesian hierarchical modelling to identify SNPs that are associated with covariates; when these covariates are environmental variables this allows for a GEA to be performed. BAYPASS is conducted on population level data, and so we used the sample PLINK population allele frequency file produced above. We first ran the core model on the genetic data alone, which produced a population covariance (omega) matrix that is used as input into the GEA models to account for covariance in genetic structure between populations due to shared ancestry. We then ran the AUX model (default settings) separately for the eight environmental variables and considered environmentally associated loci as those with a Bayesian Factor higher than 20.

### 2.6 Functional Annotation of Environmentally Associated Loci

All environmentally associated loci identified in the above analysis were then functionally annotated using the *Eucalyptus grandis* v2 genome (GCA_000612305.2) and the EUCANEXT database (Nascimento et al. 2017). For each outlier SNP, the raw sequence tag information was extracted in fasta format, and this was mapped using BLAST‐PLUS v.2.16.0 (Altschul et al. 1990, 1997; Camacho et al. 2009) against the *E. grandis* genome, with the complementary gene annotation information used to assign gene names, GO terms, and putative gene functionality to the SNPs that fell within a gene sequence or +/− 1000 bp of a gene sequence.

All environmentally associated loci were analysed together to assess whether there was a statistical enrichment for any gene ontology (GO) terms, which would point to important biological functions that may be under selection within the *E. delegatensis* mainland range. For this we used topGOv2.60.1 (Alexa and Rahnenfuhrer 2025) in *R*, testing for both biological processes and molecular function enrichment (algorithm = “weight01”, statistic = “fisher”), comparing the outlier gene set to a background gene set obtained from mapping the whole SNP dataset to the *E. grandis* genome assembly and gene annotation using BLAST‐PLUS v.2.16.0.

### 2.7 Generalized Dissimilarity Modelling of Environmentally Associated Loci

We performed generalized dissimilarity modelling (GDM) to understand how allele frequencies turnover at the environmentally associated loci changed as a function of environment. We conducted GDM analyses on all environmentally associated loci, specifically analysing each against the environmental variable for which they were flagged to be in association with. All GDM analyses was conducted in R. We used the *genet.dist* function (method = “Dch”) in the HIERFSTAT package to create a genetic distance matrix for each locus separately, before scaling all of these genetic distances by the global maximum amount (i.e., highest genetic distance found across all loci), so that all values existed on a comparable scale of 0 to 1 (this was done as GDM input must be within this range). Then in the R package GDM v1.6.0–6 (Fitzpatrick et al. 2024), we used the *formatsitepair* function to unite the genetic, environmental, and spatial (latitude and longitude) information into the appropriate format. We then passed this into *gdm* function (geo = FALSE) to identify patterns of allelic turnover with relation to environmental change, while taking into account spatial components captured by population location latitude and longitude. For loci for which a GDM model was successfully fit, we noted the null deviance explained by the GDM model (i.e., how much allelic turnover was explained by the GDM model that incorporated the environmental variable, compared to one without), as well as the l‐spline plot of allelic turnover (which captures partial genetic distance on the y‐axis by incorporating and accounting for geographic distance).

We retained and plotted only those GDM models with explained deviances above zero, and then assessed whether this result was more than would be expected by chance, by conducting random permutation subsampling (based on the total number of unique loci per environmental variable) from the full SNP dataset of 3978 loci, and running GDM models on these loci to compare how many GDM models successfully fit based on random chance, and whether the explained fit was more than would be expected by chance in the random loci subset (repeated 10 times) compared to the environmentally associated loci subset.

We tested whether the same putatively adaptive loci show independent sorting across the same environmental gradients in both subspecies. For loci that explained positive deviance for environmental variables that were identified as important by the GDM null models, we first calculated average allele frequency within each Tasmanian population. Allele frequencies were then fitted against the environmental variable values for each population to test if environmentally associated outlier loci identified as important in the mainland populations also responded to the same environmental cue within the Tasmanian subspecies. For this we used a generalized additive model approach using the function *gam* in the MGCV v1.9–4 package (Wood 2025), specifying a Gaussian distribution and applying a smoothing function to the environmental variable, with a basis dimension (*k*) of 4 to allow for a simple nonlinear (e.g., threshold or plateau) response, with latitude and longitude added as a second term in the same manner to account for spatial autocorrelation. Model overfitting was checked using the function *gam. check*.

To predict putative genomic offset across the distribution of *E. delegatensis* , we used the 13 loci with positive deviance generated from the GDM model with the best explanatory power for the loci: T_SN_, T_MIN_, T_DryQ_, P_MA_, and AHMI. Projected climate data for the 2061–2080 and 2081–2100 periods were downloaded for the CMIP6 at a 30s arc spatial resolution based on the GISS‐E2‐1‐G and at the 585 Shared Socio‐economic Pathway (SSP) that is analogous to RCP8.5, and also the 2081–2100 period also at a 30s arc spatial resolution for the GISS‐E2‐1‐G method under the less extreme 245 SSP (analogous to RCP4.5) climate scenario (NASA Goddard Institute for Space Studies 2019). To estimate putative genomic offset, we used the current climate rasterstack and GDM model and subtracted it from the projected 2081–2100 climate scenario rasterstack using the *predict* function and with *time* = TRUE, with the difference between the models with the *E. delegatensis* distribution projection.

Finally, we combined present‐day genetic data with allele turnover projected under future climate (2081–2100, SSP5‐8.5) to calculate, for each grid cell, the minimum genetic distance to its nearest neighbor across the present‐day landscape, as projected by the GDM model. Additionally, we selected one hotspot identified from this landscape projection, along with one more vulnerable (BLLA) and one less vulnerable (CAOG) population, as indicated by the genomic offset analysis, to identify which present‐day locations would act as the most suitable source populations for matching projected future climates.

## 3 Results

### 3.1 Population Structure and Diversity From Genome Wide SNPs

A total of 90,520 bi‐allelic loci were generated by DArTseq for 652 individuals across 35 populations of *E. delegatensis* , which following filtering was reduced to 5167 loci for 629 individuals across 33 populations.

Estimation of population structure across all individuals through SNMF and DAPC found strong population differentiation between the mainland and Tasmania (Figure 2A & 2C). Strong population structure was supported by both cross‐entropy analysis (Figure S2A) and Bayesian Information Criterion (BIC, Figure S2B), and thus further analysis of population structure was undertaken within each of these two range subdivisions.

**FIGURE 2:**
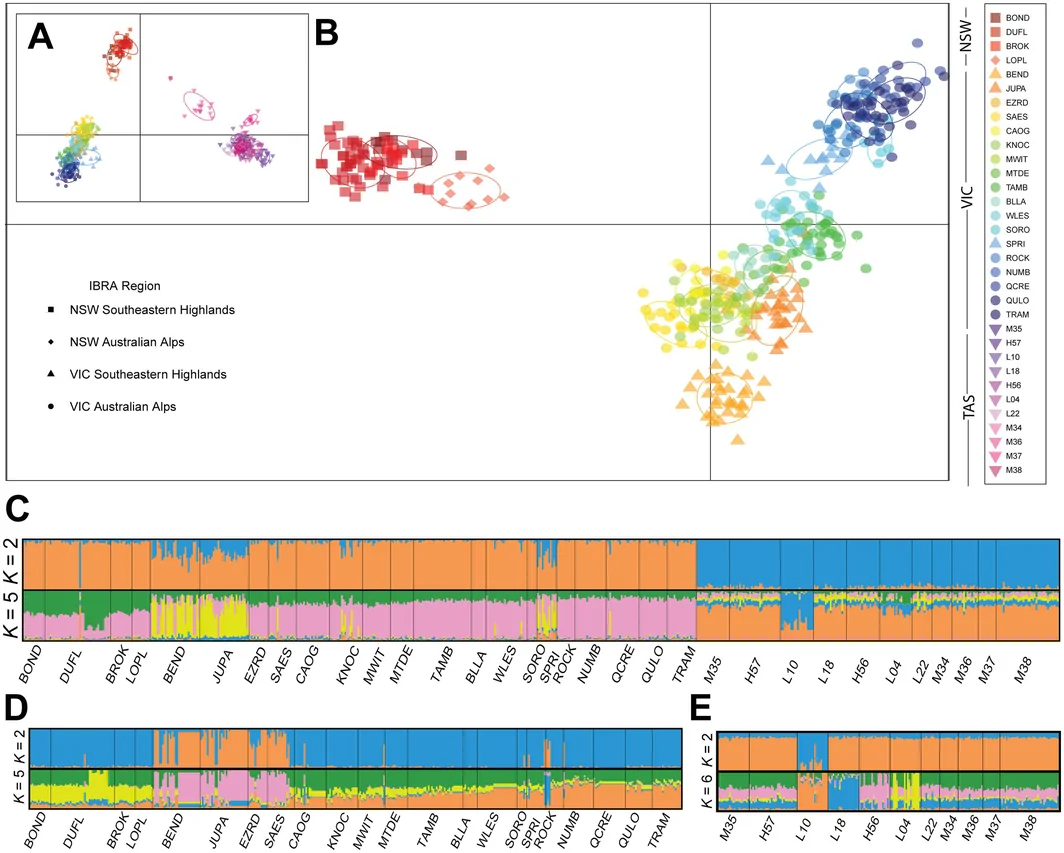
(A) all dataset and (B) mainland only dataset. Discriminant analysis of principal components (DAPC) with 225 principal components and 2 discriminant functions retained representing 68.7% of the total genetic variation (full dataset) and 165 PCs and 2 discriminant functions retained representing 67.9% total variance (mainland dataset). Ellipses around individuals and populations represent 95% CI. SNMF analysis of (C) all dataset, (D) mainland dataset, and (E) Tasmania population membership to *K* = 2 and K = 7, based on entropy genetic clusters for *E. delegatensis* where populations are orientated North to South. Note colors across separate structure plots are not necessarily equivalent to genetic ancestry. SNP counts for the full dataset, *n* = 5167; mainland, *n* = 3978; and Tasmania, *n* = 3854.

Population structure among the 22 mainland *E. delegatensis* populations from cross‐entropy analysis suggests the optimal number of genetic clusters was 5 (Figure 2D, Figure S3B), while the Bayesian Information Criterion (BIC) score suggests a *K* of 3–5 (Figure S3). DAPC analysis of these populations clearly separates the NSW populations from the VIC populations, with two genetic clusters among the VIC populations, though these are not clustered spatially. SNMF analysis does not distinguish between NSW and VIC populations when *K* = 2 but suggests the easternmost VIC populations (BEND, EZRD, JUPA, and SAES) are the most differentiated from the other *E. delegatensis* populations with some differentiation among southwestern populations (ROCK and SPRI; Figure 2C & 2D). With increasing values of *K*, no geographic clustering was visible suggesting populations are well admixed (Figure S3).

Cross‐entropy analysis also revealed strong population structure across the Tasmanian sampling locations, identifying *K* = 6 as the optimal number of genetic clusters (Figure 2E, Figure S4).

The dataset was re‐filtered to just mainland individuals, since pairwise *F*_ST_ between mainland and Tasmanian populations was > 0.157 (Table S1) with moderate population differentiation (global *F*_ST_ = 0.155). The resulting dataset was reduced to 3978 loci for 409 individuals among 22 populations. Among the mainland populations, differentiation was low (global *F*_ST_ = 0.082), with pairwise *F*_ST_ ranging from 0.016 (between KNOC and MWIT) to 0.180 (between DUFL and SPRI) (Table S1b). However, pairwise *F*_ST_ varied among populations and groups; differentiation among populations within NSW and Victoria was generally low (*F*_ST_ ≤ 0.044 between all NSW populations and ≤ 0.060 between Victorian populations), while *F*_ST_ ranged from 0.035–0.180 when comparing NSW and Victorian populations. Only three populations—BEND, JUPA and SPRI—showed consistently higher pairwise *F*_ST_ (> 0.10) when compared to other populations across NSW and Victoria (except between BEND and JUPA *F*_ST_ = 0.041, and between BEND and KNOC *F*_ST_ = 0.075; Table S1b). AMOVA of all *E. delegatensis* populations (Table 2A) shows that most variation is within individuals (57.25%, *p* = 0.001) with a large proportion of variation between regions (mainland and Tasmania, 12.50%, *p* = 0.001). AMOVA of mainland *E. delegatensis* (Table 2B) also shows that most variation lies within individuals (65.21%, *p* = 0.001), while a small proportion of the variation found between populations (6.05%, *p* = 0.001) and IBRA regions (2.48%, *p* = 0.01).

**TABLE 2:** Analysis of Molecular Variance of *E. delegatensis* populations. (A) Populations are categorized into Mainland or Tasmania regions using the full dataset of 5167 loci and (B) Populations categorized into IBRA regions (Australian Alps or South Eastern Highlands) using the mainland‐only dataset of 3978 loci.

	Df	Sum sq.	*σ*	%	*p*
**A**					
Within individuals	629	325900.00	518.124	57.25	0.001***
Among individuals within populations	596	560739.29	211.356	23.35	0.001***
Among individuals within regions	31	101987.79	62.401	6.89	0.001***
Between regions	1	68527.53	113.134	12.50	0.001***
Total	1257	1057153.61	905.015	100	
**B**					
Within individuals	409	200833.00	491.973	65.21	0.001***
Among individuals within populations	387	343072.67	197.729	26.26	0.001***
Among individuals between populations	20	50650.03	45.557	6.05	0.001***
Between IBRA regions	1	10008.22	18.652	2.48	0.01**
Total	817	604563.91	752.973	100.0	

*Note:* %, percent of the explained variation. *** denotes *p* < 0.001. ** denotes *p* < 0.01.

Abbreviations: Df, degrees of freedom; Sigma (*σ*), variance; Sum Sq, sum of squares.

### 3.2 Influence of Geographic and Environmental Distance on Genetic Differentiation

The Multiple Matrix Regression with Randomization (MMRR) analysis explained 8.7% of the variation in genetic differentiation (*R* ^2^ = 0.0867). The model coefficients indicated a positive effect of geographic distance (*β* = 0.270, *t* = 4.032, *p* = 0.046) on genetic differentiation, while environmental distance (*β* = 0.059, *t* = 0.878, *p* = 0.715) (see Table S2 for list of environmental variables) had a non‐significant effect on genetic differentiation. The overall model was borderline significant (*F* = 10.822, *p* = 0.084). Thus, within the mainland range of *E. delegatensis* , genetic differentiation between populations was moderately well predicted by geographical distance but not environmental dissimilarity.

### 3.3 Genotype‐Environment Associated Loci

The full filtered dataset of 3978 independent loci was used for each GEA approach, and putatively adaptive loci were detected for each environmental variable. The LFMM outlier analysis reported 120 outlier loci (84 unique accounting for the same SNP being flagged by multiple environmental variables) (Figure 3A). The partial redundancy analysis (RDA) (Figure 3B), controlling for spatial covariates, showed that environmental predictors explained a significant fraction of genetic variation in allele frequencies (*F* = 1.55, *p* < 0.001). The adjusted *R* ^2^ was 0.17, with the first three constrained axes (RDA1–RDA3) explaining 60% of the constrained variation and identified a total of 43 (all unique) outlier loci. These loci were then subsequently correlated with environmental variables to determine which showed the strongest associations with loci across populations (Figure S5). BayPass analysis identified the smallest amount of outlier loci, with a total of 15 unique SNPs, corresponding to 16 SNP‐environment associations (Figure S4C).

**FIGURE 3:**
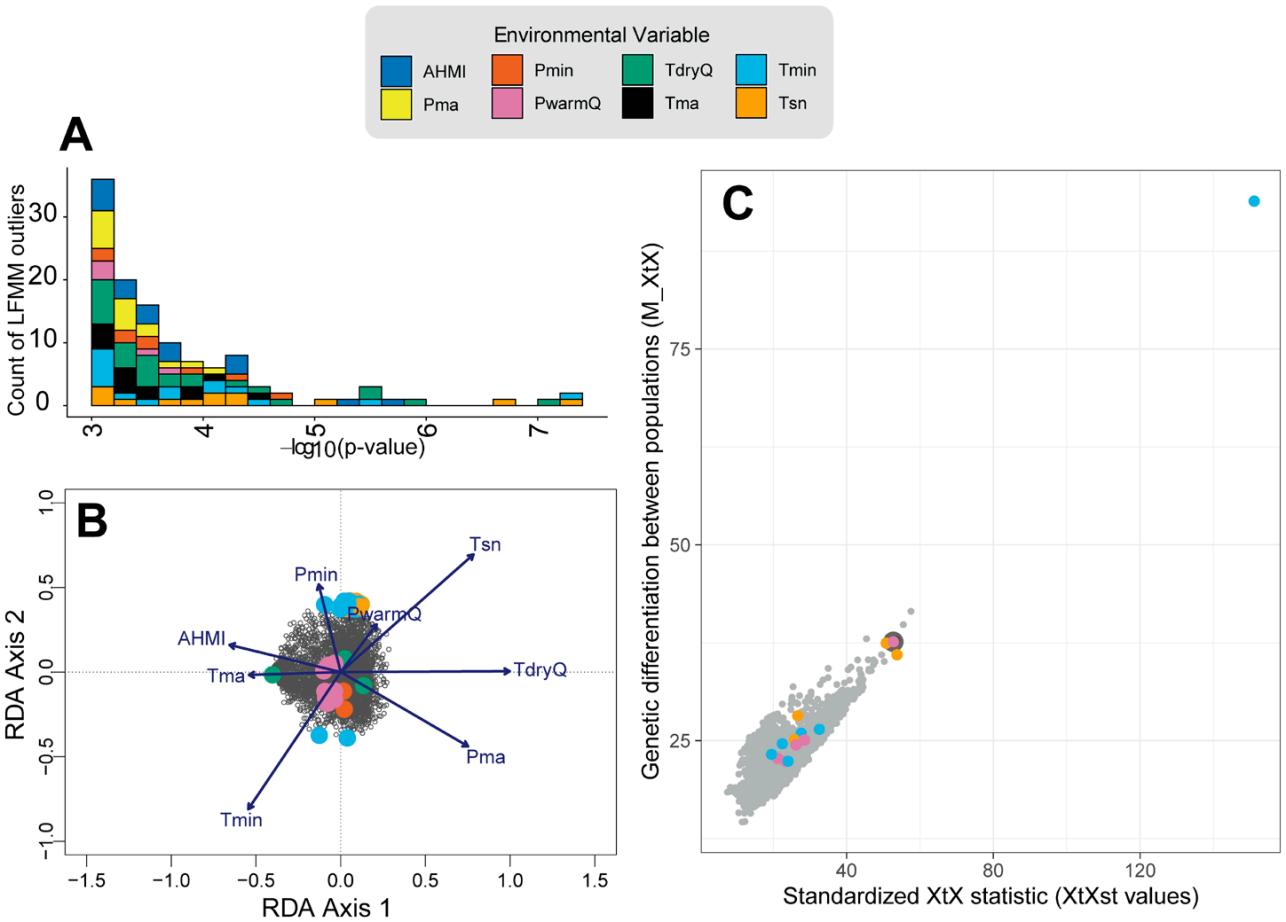
Summary of environmental association analysis for *E. delegatensis* across the three outlier detection methods. Panel (A) LFMM histogram of *p*‐values across all eight environmental variables. Panel (B) Partial redundancy analysis (RDA) showing the association between SNP variation and environmental predictors after accounting for covariates. Points represent SNPs, with color indicating the most correlated environmental variable for flagged outlier loci. Blue arrows indicate the direction and magnitude of each environmental variable. Panel (C) is a scatter plot of BayPass SNP statistics showing standardized genetic differentiation (XtXst) versus mean BayPass XtX (M_XtX) across all SNPs, with environmentally flagged outliers indicated in their respective colors. For SNPs flagged outliers by more than one environmental variable, the second environmental variable is indicated by a halo of color around the first.

Across all outlier detection methods (RDA, BayPass, and LFMM), a total of 138 unique loci were identified as candidate outliers, with only 26 of these flagged by multiple outlier methods or multiple environment variables. The number of unique loci associated with each environmental variable varied, with the highest number detected for T_MIN_ (43), T_DryQ_ (30 loci), followed by T_SN_ (25) and the lowest for P_MIN_ (11 loci) (Figure S6).

### 3.4 Functional Annotation and GO Enrichment of Environment‐Associated Loci

Of the 138 unique outlier SNPs, 71 were located in or within 1000 bp of a gene sequence when mapped to the *Eucalyptus grandis* v2.0 reference genome. For comparison, 1892 of the 3978 mainland population‐genomic SNPs in our dataset were similarly associated with a gene sequence, providing the background set for enrichment analysis.

Gene Ontology enrichment of the outlier‐associated genes identified several processes and functions potentially relevant to adaptation across these sub‐alpine environments. Across biological processes and molecular functions, a total of 10 GOs were enriched at a *p* ≤ 0.05 significance threshold, and a further 11 at 0.05 < *p* ≤ 0.1. For biological process, the most significantly enriched term was cellular response to DNA damage stimulus (GO:0006974, *p* = 0.045) (Table S3), and for molecular function was 2‐oxoglutarate‐dependent dioxygenase activity (GO:0016706, *p* = 0.0067).

### 3.5 Landscape Genomics of Genotype‐Environment Associations

All 138 unique loci identified by the GEA methods associated with the eight environmental variables were placed into a GDM (Figure S6). Of the 19 loci associated with P_WarmQ_, the 15 loci associated with T_MA_, and the 11 loci associated with P_MIN_, we observed no loci exhibiting allelic turnover across environmental space as would be indicated by a significant GDM model. All other environmental variables showed allelic turnover (Figure 4A): T_MIN_ (7/41 outlier loci), T_SN_ (5/23 outlier loci), T_dryQ_ (6/30 outlier loci), P_MA_ (3/16 outlier loci) and AHMI (1/19 outlier loci).

**FIGURE 4:**
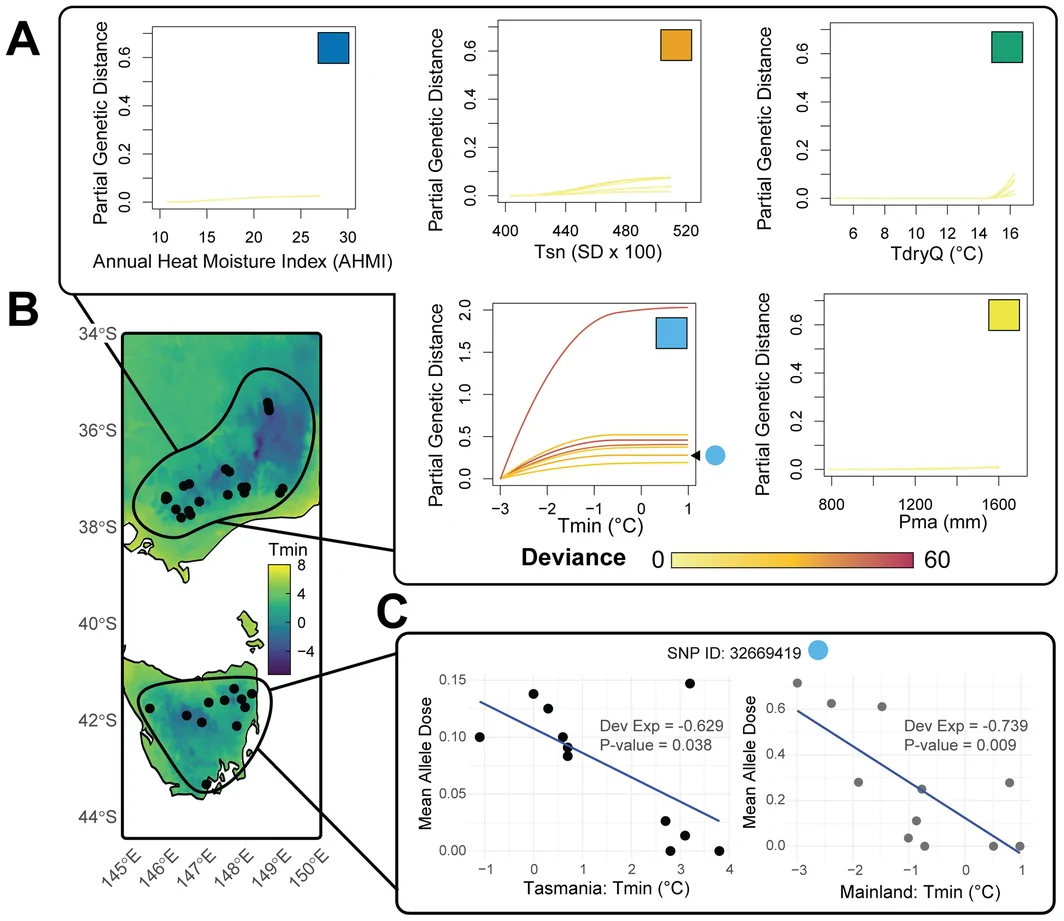
Panel (A) partial genetic distances for outlier SNPs with successfully fitted GDMs along their corresponding environmental gradients. Each line represents a fitted spline for a single SNP, colored by the proportion of deviance explained (yellow = low, orange = moderate, red = high). Only AHMI, P_WarmQ_, P_MA_, T_MIN_, T_dryQ_ and T_SN_ are shown, as these were the only environmental variables for which a GDM could be fit. Predicted allelic turnover across environmental space illustrates how each variable contributes to genetic differentiation among populations and across environmental gradients. Coloured squared within each panel indicates the color scheme used for each on previous figure plots. Panel (B) is a map of populations sampled for this study, indicating which samples went into the GDM models in Panel (A) and which were reinvestigated in panel (C). Panel (C) allele frequencies for a subset of T_MIN_‐associated outlier SNPs across independent Tasmanian populations (with the same plot for the mainland populations presented on the right as a comparison). The green square indicates which loci returned a significant correlation between allele dose and T_MIN_ in Tasmania.

The level of allelic turnover (partial genetic distance) differed between each of the five environmental variables, though patterns of turnover remained largely consistent for all loci tested against each specific environmental variable. For T_SN_, the allelic turnover exhibited a gradual cline, while for T_dryQ_ it showed rapid observed turnover across environmental space at the high end of the scale. For T_MIN_, the only environmental variable that showed strong and consistent explained deviance values, allelic turnover rapidly increased until −1°C and then plateaued. Across all environments, most loci exhibited a low (< 0.1) allelic turnover; however, for T_MIN_, all loci that had a higher turnover (> 0.3) (Figure 4A). The total deviance explained was higher than expected under a null distribution for T_MIN_, T_SN_, and T_dryQ_ (Table S4), whereas the total number of outlier SNPs for which a GDM model could be successfully fitted was elevated for all but not AHMI (Table S5).

We then reinvestigated the allele frequency relationship with the environmental variable for the seven loci identified as having positive deviance for T_MIN_, as this was the only environmental variable found to have passed both the null distribution GDM models and where the loci displayed high allelic turnover and explained deviance. While two of these loci did not have the alternate allele present in the Tasmanian population, the population allele frequencies for the remaining five loci were correlated against their populations' T_MIN_ values. One locus was found to have a significant association with T_MIN_ (Figure 4C). Intriguingly, these were not the loci with the highest deviance in the mainland populations (Figure 4A, T_MIN_ plot).

The projected genomic vulnerability was estimated using 19 loci with positive deviance from the GDMs (explaining 46.63% of the variation) under a 2081‐2100 ssp585 climate model (Figure 5A), a 2061‐2080 ssp585 climate model (Figure 5B), and a 2081‐2100 ssp245 climate model (Figure 5C). Under each future climate scenario, populations within the central region of the mainland *E. delegatensis* distribution throughout Victoria may be vulnerable to projected climate change (dark red), while populations at the range edges may not be as severely impacted. Although in the less extreme climate scenario, range‐edge populations in the northeast (New South Wales) may be vulnerable (Figure 5C). Southeastern Victoria range‐edge populations (BEND and JUPA) consistently appear to be least impacted across each future‐climate scenario. These populations at the southeastern range edge in Victoria (BEND and JUPA) also exhibit higher heterozygosity among these loci than other Victorian and New South Wales populations (Figure 5D), though moderate heterozygosity (0.10–0.15) of the 19 loci is observed among populations both in the central distribution in Victoria and range‐edge New South Wales.

**FIGURE 5:**
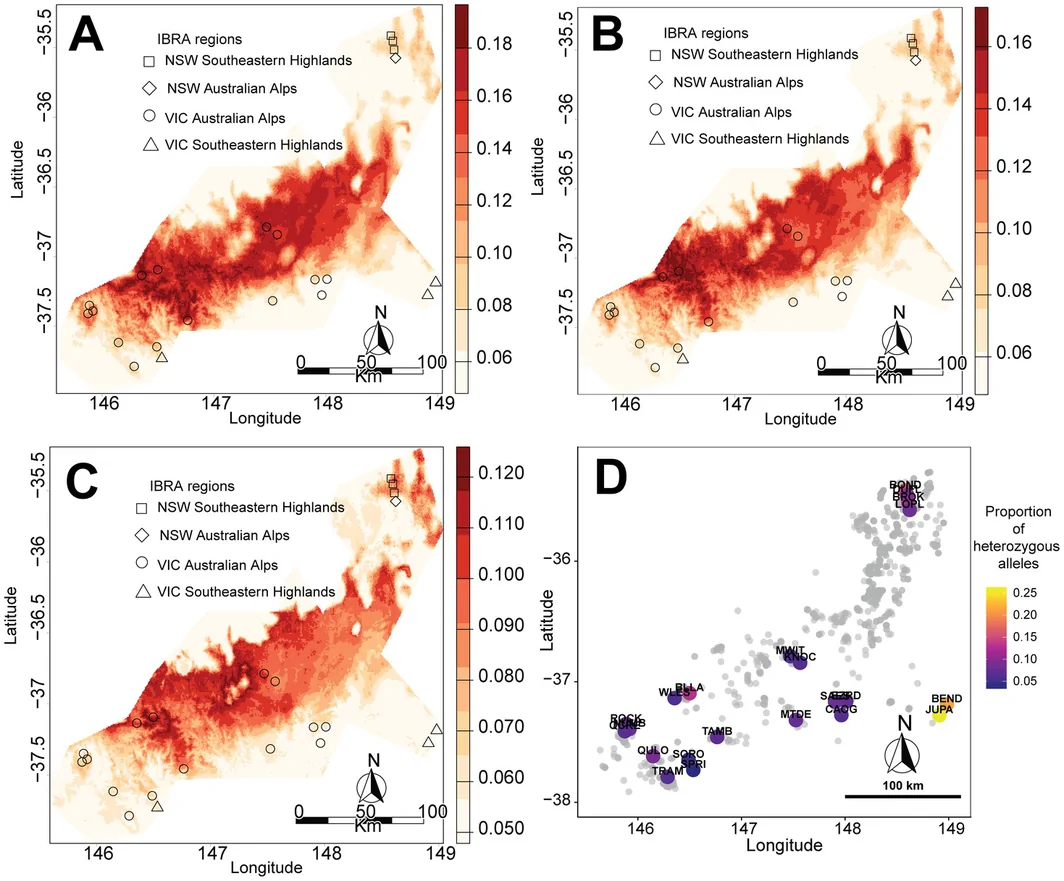
(A) Projected genomic vulnerability for a 2081‐2100 ssp585 climate scenario across the mainland *E. delegatensis* distribution using the 19 loci with positive deviance resulting from the GDM model; (B) projected genomic vulnerability across the *E. delegatensis* distribution for a 2061‐2080 ssp585 climate scenario with the 19 loci with positive deviance; (C) projected genomic vulnerability for a 2081‐2100 ssp245 climate scenario across the *E. delegatensis* distribution using the same 19 loci. Symbols in A–C denote differences in IBRA region between populations and (D) the heterozygosity of these loci among the mainland *E. delegatensis* populations. Grey dots represent species occurrence data collected from Atlas of Living Australia (ALA).

We found that much of the species' mainland range currently contains populations suitable as source populations for climate‐adjusted seed (i.e., seed matched to projected future climates, using our GDM model with positively deviant climate‐associated loci). Genetic distance to the nearest neighbour did increase in some regions, particularly at higher elevations, highlighting potential populations for seed sourcing (Figure 6A). When we selected three diverse focal populations for which we projected genetic matches between the present‐day range and future climate, no single source population was ideal across all three examples. In general, however, when projecting sourcing for more alpine/higher‐elevation populations, the northernmost populations tended to be better genetic matches, while the lowland, southern populations formed a distinct source region of their own (Figure 6B–D).

**FIGURE 6:**
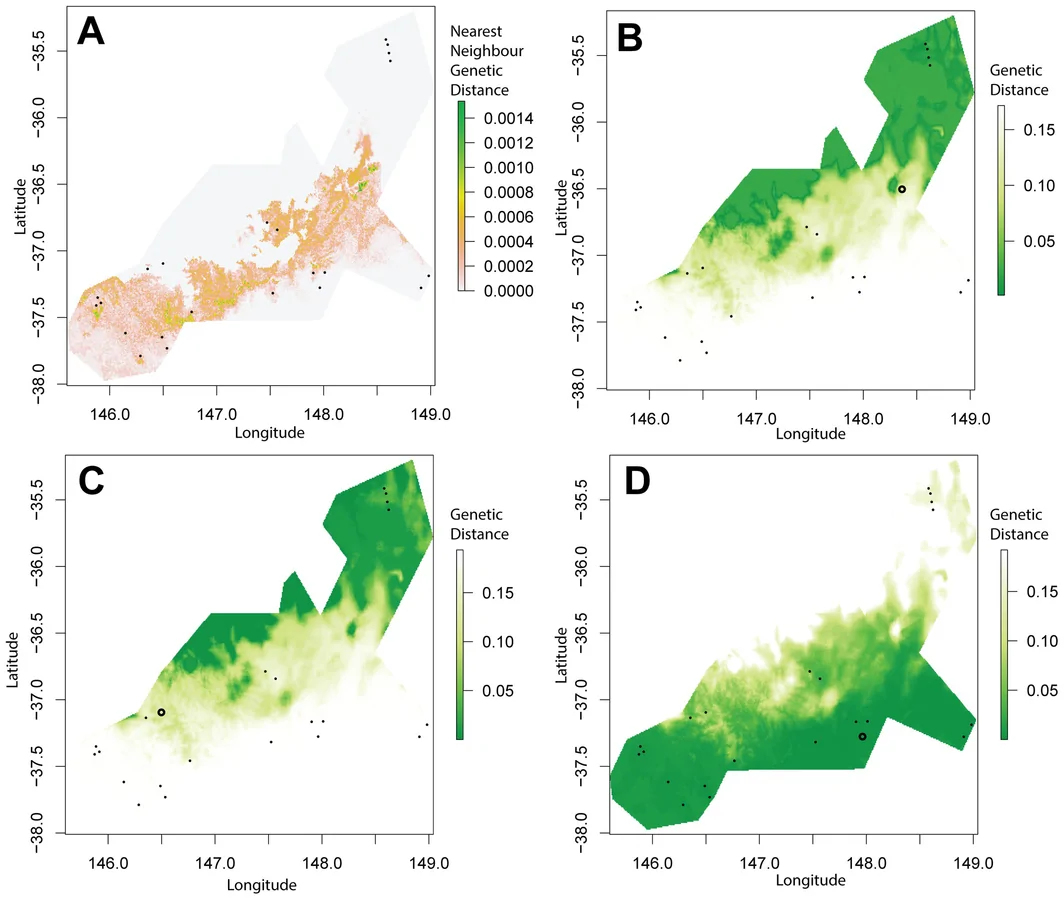
(A) Genetic distance between a location's ideal genetic profile under the future climate scenario (2081‐2100 ssp585) and its single best current‐day match across the mainland *E. delegatensis* distribution, as informed by the GDM model. Low genetic distance means that a genetically matching present day population is available as a potential genetic source to help future proof a region, where regions with higher nearest neighbour genetic distance have less ideal future climate informed genetic matches. (B–D) the genetic distance between present day potential genetic sources in relation to a focal population; (B) the region with the highest nearest neighbour genetic distance as indicated in (A); (C) location BLLA, which is projected to have high genomic vulnerability across all models in Figure 5; and COAG that is predicted to have less genomic vulnerability across all models in Figure 5.

## 4 Discussion

Our study has identified and quantified putative adaptive genetic diversity in the keystone Australian alpine species *Eucalyptus delegatensis* . We have found that environmental adaptation in this species is likely to be polygenic and complex, controlled by many loci and/or genes of small effect, unsurprising given that similar results have been observed for plant traits and genomic analyses among tree species (De La Torre et al. 2021; de Miguel et al. 2022; Porth et al. 2015; Sork et al. 2016), eucalypts (Ahrens et al. 2019; Felipe et al. 2022; Jordan et al. 2017; Rocha et al. 2022; Steane et al. 2017) and alpine plants (Minadakis et al. 2024; Szukala et al. 2023; Zacharias et al. 2022). Many species exhibit these trends along latitudinal gradients that are strongly associated with temperature, however for *E. delegatensis* we observe these along both latitudinal and elevational temperature gradients. Among other eucalypts that have a latitudinal distribution (e.g., *Corymbia calophylla*, Ahrens et al. 2019; *E. marginata* , Felipe et al. 2022; *E. melliodora* , Supple et al. 2018; *E. microcarpa* , Jordan et al. 2017), populations in the northern distribution generally exhibit warmer temperatures, however for *E. delegatensis* , northern populations generally experience moderate mean annual and summer temperatures, and the coldest winter temperatures.

### 4.1 Genomic Signatures of Climate Adaptation

Climate and environmental variables can drive local adaptation in trees through standing genetic variation on polygenic traits. Allelic shifts across environmental gradients among *E. delegatensis* populations were generally low, as have been observed among other eucalypts (Ahrens et al. 2019; Buttler et al. 2022; Sork et al. 2016). While of low effect, these loci may be associated with molecular pathways involved in ion uptake, gene expression regulation and stress response. Despite this, of the nineteen positively deviant loci within the future climate projection, seven are associated with T_MIN_ (BIO06), with five of these loci exhibiting an allelic shift greater than 0.3. This was expected due to the sub‐alpine environments, thus reinforcing that temperature is an important abiotic driver of selection in this species. We found some of these loci align to genes (X0117, nitrate transport 1.1) or gene ontologies (X4092, oxidation–reduction process) that have been linked with plant growth and stress responses (Farrow and Facchini 2014; Rathinasabapathi et al. 1997; Ro et al. 2005; Shimada et al. 2001), while others were not aligned with a genic region (Table S3). In these cases, the loci may be linked to other regions in the genome that may be of adaptive importance in this species. Cold‐acclimated Australian plants are known to be better at regulating respiration and photosynthesis improving carbon gain, and increased nitrogen and non‐structural carbohydrate accumulation, including *E. delegatensis* (Campbell et al. 2007). These responses could be important under climate change scenarios, particularly under climate extreme events (e.g., heatwaves, droughts and floods), that may mitigate the impacts of environmental stressors. Intra‐specific variation of these traits may exist throughout the *E. delegatensis* distribution and should be investigated to identify potentially vulnerable provenances.

### 4.2 Projected Vulnerability in Future Climates

Our data suggest that central mainland *E. delegatensis* populations may be most vulnerable under all our projected climate change scenarios, with vulnerability increasing with the severity and temporal distance of the projected scenario (Figure 5A–C). If these central populations (BLLA, KNOC, MWIT, TAMB, and WLES) should experience a reduction in fitness, it also coincides with low‐to‐moderate heterozygous alleles that may also impact their ability to adequately remain productive through climate change. Populations at the southeastern distribution limits (e.g., BEND, JUPA) were among those projected to be least impacted, and also exhibit higher heterozygosity, consistent with greater adaptive capacity, as has been reported for other range‐ or niche‐edge plant populations (Edgeloe et al. 2022; Halbritter et al. 2015; Pennington et al. 2021; Rehm et al. 2015). However, elevated heterozygosity at range edges does not necessarily indicate high adaptive potential, as similar patterns can arise through allele surfing (Edmonds et al. 2004; Hallatschek et al. 2007; Hallatschek and Nelson 2010). This is unlikely to explain our results, however, because *F*_IS_ values of these populations are comparable to those in the rest of the range, suggesting no strong demographic signatures associated with range expansions. Under both moderate and extreme projections, all populations are projected to experience warming, with T_MIN_ increasing to ~0°C in almost all projections, alongside variable precipitation changes (Figure S7, S8). These changes may impact population productivity, particularly if water accessibility is reduced and compounded with extreme climatic events such as droughts, that are expected to occur in Australia and in sub‐alpine environments (Chapman et al. 2025; Lesk et al. 2022; Matusick et al. 2018). Despite this vulnerability, our seed‐sourcing projections (Figure 6) indicate that suitable genetic matches for most populations' projected future climates remain available elsewhere within the mainland range. This may provide a means for assisted gene flow to allow populations to keep pace with shifting future climates, as has been applied in other long‐lived tree species (e.g., MacLachlan et al. 2018).

However, while some regions are projected to have lower future climate vulnerability, confounding abiotic factors, including increased fire activity and/or intensity, may negate the benefit of climate adjusted adaptive variation within these populations. Fire is a common feature of heatwaves in these environments, and is projected to increase in frequency, intensity, and duration in the future (IPCC, 2021). Since *E. delegatensis* is fire intolerant and an obligate seeder, increased fire risk is projected to be a serious threat to mature trees and saplings that may not have reached maturity, even forcing populations to become locally extinct (Hammond et al. 2026). While this species' obligate seeding strategy is well suited to the historically infrequent, high‐intensity fires of its sub‐alpine range, the increasing frequency of such fires under anthropogenic climate change may outpace the speed at which seeds mature and replenish the population and the seed bank. Given this uncertainty, population resilience will undoubtedly be shaped by how fire is actively managed within this landscape and identifying viable seed source options for most at risk populations will be a necessary precautionary measure, regardless of their currently projected genomic resilience.

*E. delegatensis* populations may also exhibit adaptive plasticity with limited or no loss of fitness. Physiological trade‐offs may also occur, including reduced photosynthetic efficiency, as observed in other alpine tree species (Neuner and Buchner 2023). Notably, the central region of the distribution exhibits the greatest difference between the current and projected climate within the GDM models. However, these populations may not be the most vulnerable when factors beyond genomic signals are considered, highlighting the importance of empirical studies and common gardens to validate these predictions. Such studies are currently underway for *E. delegatensis* , and have so far identified limited population differences in drought tolerance under current common garden conditions (Gurung et al. 2025); additional traits and future‐climate treatments are needed to more directly test the vulnerability patterns suggested by genomic offset.

### 4.3 Cold and Frost Tolerance Under Climate Change

*Eucalyptus delegatensis* , like other alpine species, is acclimated to cold temperatures and frost (Neuner 2014) with taller trees generally experiencing a reduced risk of frost‐related injuries (Körner 2012). *Eucalyptus* tree species have also been observed to reduce photosynthetic output following frost events (Davidson et al. 2004), that may also limit impacts of frost injury. These tolerances and responses to cold and frost are unsurprising, particularly for *E. delegatensis* , since seed germination can occur at 2°C and cold stratification of seed improves germination success (Battaglia 1993) particularly among provenances from higher altitudes (Close and Wilson 2002). Under climate change however, *E. delegatensis* trees may produce fewer and smaller seed as has been observed in the Australian alpine‐subalpine herbaceous species *Wahlenbergia ceracea* (Notarnicola et al. 2023). However, if germination success is impaired in *E. delegatensis* , populations may experience declines or mortality, particularly following extreme disturbance events. While climate projections suggest temperatures will be warmer, this does not eliminate extreme cold events, though these events may become increasingly infrequent and shorter in duration. Should this be the case, climate warming and the limitation of extreme cold events may impact the germination success of *E. delegatensis* , particularly if cold events are short in duration.

Repeated exposure to cold and frost may also have important consequences for mature trees. Trees can deacclimate to cold and frost upon the onset of spring (Bokhorst et al. 2010; Buchner and Neuner 2011), potentially increasing the risk of frost‐related injury and mortality, particularly following an early snow melt (Wheeler et al. 2014). Under warmer winters with repeated extreme frost events, *E. delegatensis* may therefore draw upon carbohydrate reserves to mitigate the impacts of stress and remain productive. Depletion of these reserves may limit the ability of plants to flower, especially later in the growing season. Warmer winters may also alter flowering phenology, with higher elevation provenances flowering earlier. Such shifts could reduce pollination success if pollinator phenology does not change concurrently. They could also alter flowering synchrony between provenances, which may reduce interpopulation gene flow. Further studies of flowering time, pollinator activity and gene flow among *E. delegatensis* populations would therefore help inform future management strategies.

### 4.4 Population Structure and Its Conservation Implications

Intriguingly, one of the positively deviant T_MIN_ loci that was assessed for parallel environmental association in Tasmanian *E. delegatensis* range also showed a significant relationship between allele frequencies and T_MIN_ across the Tasmanian sampling locations (Figure 4C) despite the strong population differentiation from the mainland. This pattern is consistent with adaptation from standing genetic variation, inherited from shared ancestral populations (Battlay et al. 2025; Chaturvedi et al. 2022; Konečná et al. 2021). Natural selection may have therefore independently sorted ancestral variants along climatic gradients on the mainland and Tasmania, resulting in repeated adaptation to T_MIN_ at this locus across geographically isolated parts of this species' range.

Population structure of *Eucalyptus delegatensis* was more strongly associated with geographic proximity than with general environmental similarity, although minimum temperature of the coldest month (T_MIN_) also emerged as an important environmental correlate. We found that population structure detection methods generally clustered populations by IBRA regions and populations in Victoria clustered along a northeast to southwest axis under some metrics. This result contrasts with a comparative analysis across over 100 species which showed a lack of clustering by IBRA regions in the majority of plant species across southeastern Australia (Fahey et al. 2024). However, few of those species were located across heterogeneous sub‐alpine environments characteristic of *E. delegatensis* . Other measures of population clustering and differentiation in *E. delegatensis* showed environmental distance did not predict general patterns of genetic differentiation within the mainland range. This highlights the value of complementary approaches to partitioning genetic variation, as different methods capture different components of genetic variation and may vary in their sensitivity to underlying differentiation. More broadly, weak correspondence between genetic variation and environmental differentiation is not unusual (Sexton et al. 2014).

Several factors could contribute to weak correspondence between genetic variation and environmental variables. The realized ecological niche of *E. delegatensis* may be is more specific than the set of environmental variables used in our analysis to represent environmental distance. However, several biological explanations are also plausible. Alternatively, cryptic barriers to gene flow or very strong selection under local climate conditions could be driving the distinctly different population signatures seen in some regions via SNMF (Figure 2B & Figure 2D). *Eucalyptus* species also exhibit extensive intraspecific structural variability which may contribute to or reflect barriers to gene flow (Ferguson et al. 2024b, 2024a). Structural variants can also facilitate parallel adaptation (e.g., Battlay et al. 2025), raising the possibility that they contribute to the parallel environmental associations we observed across mainland and Tasmanian populations. However, we did not directly assess structural variation in this study, and such complex genomic architecture is unlikely to be fully captured by the reduced‐representation approaches used in this study. Future work should therefore apply whole‐genome resequencing, ideally supported by a species‐specific reference genome, to better characterize the full spectrum of genetic variation in this species and to investigate the mechanisms underlying these patterns.

Together, these results demonstrate that geography provides a useful but incomplete picture of population and adaptive variation in *E. delegatensis* . This distinction has direct practical relevance for seed sourcing under climate change: although geographically proximate populations may often represent suitable sources, our projections also indicate that the geographically nearest population is not always the best‐matched genetic source for a given site's projected future climate.

## Funding

This project was funded by Greening Australia.

## Conflicts of Interest

The authors declare no conflicts of interest.

## Supporting information


**Table S1:** eva70339‐sup‐0001‐DataS1.docx.
**Table S2:** eva70339‐sup‐0001‐DataS1.docx.
**Table S3:** eva70339‐sup‐0001‐DataS1.docx.
**Table S4:** eva70339‐sup‐0001‐DataS1.docx.
**Table S5:** eva70339‐sup‐0001‐DataS1.docx.
**Figure S1:** eva70339‐sup‐0001‐DataS1.docx.
**Figure S2:** eva70339‐sup‐0001‐DataS1.docx.
**Figure S3:** eva70339‐sup‐0001‐DataS1.docx.
**Figure S4:** eva70339‐sup‐0001‐DataS1.docx.
**Figure S5:** eva70339‐sup‐0001‐DataS1.docx.
**Figure S6:** eva70339‐sup‐0001‐DataS1.docx.
**Figure S7:** eva70339‐sup‐0001‐DataS1.docx.
**Figure S8:** eva70339‐sup‐0001‐DataS1.docx.

## Data Availability

The data that support the findings of this study are freely available on Zenodo.org, DOI: https://www.zenodo.org/records/23753938.
