# Supplementary material for: Adaptive Genomic Variation Across Sub‐Alpine Climates in a Keystone Forest Tree: Implications for Seed Sourcing for Restoration in a Changing Climate
Source: Evol Appl. 2026 Nov 6;19(11):e70339. doi: 10.1111/eva.70339 (PMC13743865; doi:10.1111/eva.70339)
Supplement: Supplementary file 1 [file EVA-19-e70339-s001.docx]

Adaptive genomic variation across sub-alpine climates in a keystone forest tree: implications for seed sourcing for restoration in a changing climate

**Supplementary Material**

Table S1a: Pairwise *F*_ST_ between populations of *E. delegatensis* (mainland and Tasmania) to a 0.05 significance threshold. Orange cells represent higher pairwise *F*_ST_ values, blue cells represent lower pairwise *F*_ST_ values. Populations are arranged from northeast to southwest among the mainland populations. See Table 1 and Figure 1 for population codes and descriptions.

Table S1b: Pairwise *F*_ST_ as in Table S1a but only mainland populations of *E. delegatensis* to a 0.05 significance threshold. Orange cells represent higher pairwise *F*_ST_ values, blue cells represent lower pairwise *F*_ST_ values. Populations are arranged from northeast to southwest. See Table 1 and Figure 1 for population codes and descriptions.

Table S2: Pearson’s correlation coefficient (r) of the 19 bioclimatic variables from WorldClim2 (Fick & Hijmans, 2017) for the distribution of *E. delegatensis*. Highlighted cells represent higher correlation (> ±0.7) between pairwise environmental variables. T_MA_, mean annual temperature; MDR, mean diurnal range; ISO, isothermality; T_SN_, temperature seasonality; T_MAX_, mean temperature of the warmest month; T_MIN_, mean temperature of the coldest month; T_AR_, mean temperature annual range; T_WetQ_, mean temperature of the wettest quarter; T_DryQ_, mean temperature of the driest quarter; T_WarmQ_, mean temperature of the warmest quarter, T_ColdQ_, mean temperature of the coldest quarter; P_MA_, mean annual precipitation; P_MAX_, mean precipitation of the wettest month; P_MIN_, mean precipitation of the driest month; P_SN_, precipitation seasonality; P_WetQ_, mean precipitation of the wettest quarter; P_DryQ_, mean precipitation of the driest quarter, P_WarmQ_, mean precipitation of the warmest quarter, P_ColdQ_, mean precipitation of the coldest quarter.

**Table S3:** Gene Ontology (GO) enrichment results showing biological processes and molecular functions that are overrepresented among outlier loci that were overlapping protein coding sequences, or within 1,000bp of it. The table includes the GO identifier, the description of the GO term, the number of genes in the background set annotated with that term, the number of genes in the tested set annotated with the term (outlier loci), the number of genes expected to be annotated by chance, the enrichment significance *p*-value from the Fisher’s exact test. Results with a *p*-value of <0.1 are all displayed.

| **GO ID** | **Term** | **Annotated** | **Significant** | **Expected** | **P-Value** |
| --- | --- | --- | --- | --- | --- |
| **Biological Process** | | | | | |
| GO:0006974 | cellular response to DNA damage stimulus | 10 | 2 | 0.46 | 0.045 |
| GO:0010215 | cellulose microfibril organization | 1 | 1 | 0.05 | 0.046 |
| GO:0016049 | cell growth | 1 | 1 | 0.05 | 0.046 |
| GO:0006284 | base-excision repair | 1 | 1 | 0.05 | 0.046 |
| GO:0048478 | replication fork protection | 1 | 1 | 0.05 | 0.046 |
| GO:0006406 | mRNA export from nucleus | 1 | 1 | 0.05 | 0.046 |
| GO:0045893 | positive regulation of DNA-templated transcription | 1 | 1 | 0.05 | 0.046 |
| GO:0016567 | protein ubiquitination | 9 | 2 | 0.41 | 0.060 |
| GO:0006741 | NADP biosynthetic process | 2 | 1 | 0.09 | 0.089 |
| GO:0006662 | glycerol ether metabolic process | 2 | 1 | 0.09 | 0.089 |
| GO:0006353 | DNA-templated transcription termination | 2 | 1 | 0.09 | 0.089 |
| **Molecular Function** | | | | | |
| GO:0016706 | 2-oxoglutarate-dependent dioxygenase activity | 10 | 3 | 0.42 | 0.0067 |
| GO:0051082 | unfolded protein binding | 1 | 1 | 0.04 | 0.0418 |
| GO:0016708 | oxidoreductase activity, acting on paired donors, with incorporation or reduction of molecular oxygen, NAD(P)H as one donor, and incorporation f two atoms of oxygen into one donor | 1 | 1 | 0.04 | 0.0418 |
| GO:0004252 | serine-type endopeptidase activity | 9 | 2 | 0.38 | 0.0511 |
| GO:0005515 | protein binding | 228 | 15 | 9.54 | 0.0592 |
| GO:0004842 | ubiquitin-protein transferase activity | 10 | 2 | 0.42 | 0.0622 |
| GO:0003951 | NAD+ kinase activity | 2 | 1 | 0.08 | 0.0819 |
| GO:0015299 | solute:proton antiporter activity | 2 | 1 | 0.08 | 0.0819 |
| GO:0003713 | transcription coactivator activity | 2 | 1 | 0.08 | 0.0819 |
| GO:0051537 | 2 iron, 2 sulfur cluster binding | 2 | 1 | 0.08 | 0.0819 |

**Table S4**: Null model comparison of GDM results (total deviance explained for each ENV variable). Summary of total deviance explained by SNP–environment GDM models compared with neutral expectations generated from 10 random‐SNP permutations per environmental variable. For each environmental predictor (Env), the table reports the total deviance explained by the real SNP set (real_total), the mean of the null permutation distribution (null_mean), and the 95% null interval (null_2.5, null_97.5). real_above_null indicates whether the observed total deviance exceeds the upper 97.5% quantile of the null distribution, and thus whether the genomic signal is stronger than expected under neutrality. Note negative deviances were set to 0, as negative values arise from mathematical artefacts in GDM and do not reflect biologically meaningful negative contributions.

| **env** | **real_total** | **null_mean** | **null_2.5** | **null_97.5** | **real_above_null** |
| --- | --- | --- | --- | --- | --- |
| AHMI | 1.344145 | 2.680322 | 0.179903 | 7.395595 | FALSE |
| P_MA_ | 0.622229 | 2.43785 | 0.420963 | 4.534784 | FALSE |
| T_DryQ_ | 10.77216 | 3.80644 | 0.14373 | 9.169776 | TRUE |
| T_MIN_ | 271.1725 | 9.500727 | 0.13604 | 27.99721 | TRUE |
| T_SN_ | 14.72912 | 2.16631 | 0.076133 | 9.987033 | TRUE |

**Table S5**: Null model comparison of GDM results (total count of deviant SNPs identified for each ENV variable). Summary of total deviance explained by SNP–environment GDM models compared with neutral expectations generated from 10 random‐SNP permutations per environmental variable. For each environmental predictor (Env), the table reports the total number of deviant SNPs identified for the real SNP set (real_total), the mean of the null permutation distribution (null_mean), and the 95% null interval (null_2.5, null_97.5). real_above_null indicates whether the observed total deviance exceeds the upper 97.5% quantile of the null distribution, and thus whether the genomic signal is stronger than expected under neutrality.

| **env** | **real_count** | **null_mean** | **null_2.5** | **null_97.5** | **real_above_null** |
| --- | --- | --- | --- | --- | --- |
| AHMI | 1 | 2 | 1 | 3 | FALSE |
| P_MA_ | 3 | 1.666667 | 1 | 2.9 | TRUE |
| T_DryQ_ | 6 | 1.857143 | 1 | 2.85 | TRUE |
| T_MIN_ | 7 | 1.9 | 1 | 3 | TRUE |
| T_SN_ | 5 | 1.8 | 1 | 3 | TRUE |

Figure S1: Estimates of kinship among the mainland *E. delegatensis* populations.

**Figure S2**: All sites dataset (A), cross-entropy plot against the number of ancestral populations and (B), the BIC against number of genetic clusters, both methods can be indicative of the number of genetic clusters within a dataset.

**Figure S3**: Mainland dataset (A), Full Sparse Non-negative Matrix Factorization (SNMF) plots for the mainland dataset (B) cross-entropy plot against the number of ancestral populations and (C), the BIC against number of genetic clusters, both methods can be indicative of the number of genetic clusters within a dataset.

**Figure S4:** Tasmanian dataset (A) Full Sparse Non-negative Matrix Factorization (SNMF) plots and (B) cross entropy outputs

**Figure S5:** Partial redundance environmental correlations with outlier SNPs, with panel (a) the strongest SNP–environment correlations (highest correlation per SNP) and panel (b) correlations across all candidate loci – environmental variables (all correlations per SNP).

**Figure S6:** A: Summary of environmental association analysis for *E. delegatensis*. Venn diagrams show the intersections between the two genotype-environment association (GEA) approaches (LFMM, partial RDA, and Baypass) and the number of putatively adaptive loci detected within and across methods for eight environmental variables: T_MA_, mean annual temperature; T_SN_, temperature seasonality; T_MIN_, mean minimum temperature of the coldest month; T_DryQ_, mean temperature of the driest quarter; P_MA_, mean annual precipitation; P_MIN_, mean minimum precipitation of the driest month; P_WarmQ_, mean precipitation of the warmest quarter; and AHMI, mean annual heat moisture index.

**Figure S7:** Future climate changes for each mainland *E. delegatensis* population under the moderate ssp245 climate scenario for the time period 2081-2100 under the GISS-E2-1-G method. Red points and lines denotes increased changes in temperature, precipitation, or AHMI, blue points and lines denotes decreased changes, and black points denotes no change. T_MA_, mean annual temperature; T_SN_, mean temperature seasonality; T_MIN_, mean minimum temperature of the coldest month; T_DryQ_, mean temperature of the driest quarter; P_MA_, mean annual precipitation; P_MIN_, mean minimum precipitation of the driest month; P_WarmQ_, mean precipitation of the warmest quarter; and AHMI, mean annual heat moisture index.

**Figure S8:** Future climate changes for each mainland *E. delegatensis* population under the more extreme ssp585 climate scenario for the time period 2081-2100 under the GISS-E2-1-G method. Red points and lines denotes increased changes in temperature, precipitation, or AHMI, blue points and lines denotes decreased changes, and black points denotes no change. T_MA_, mean annual temperature; T_SN_, mean temperature seasonality; T_MIN_, mean minimum temperature of the coldest month; T_DryQ_, mean temperature of the driest quarter; P_MA_, mean annual precipitation; P_MIN_, mean minimum precipitation of the driest month; P_WarmQ_, mean precipitation of the warmest quarter; and AHMI, mean annual heat moisture index.
